# Supplementary material for: Content-rich biological network constructed by mining PubMed abstracts
Source: BMC Bioinformatics. 2004 Oct 8;5:147. doi: 10.1186/1471-2105-5-147 (PMC528731; doi:10.1186/1471-2105-5-147)
Supplement: Additional File 2 — The original results of the above study (non-essential files are deleted to keep the file size under the limit set by BMC bioinformatics). [file 1471-2105-5-147-S2.bz2 › chilibotAdditionalFile2/dip05/514ID8799122E10817/html/P300_ESR1.html]

 


 **P300** and **ESR1** 
  
Found 50 abstracts in PubMed, retrieved 05.  
 

 What does Google say? 
 PDF only 
| .edu only 

---

**Interactive relationship** (e.g. stimulation, inhibition, etc)

**Neutral relationship**- pRb2 p130 E2F4 5 HDAC1 SUV39H1  **p300**  and pRb2 p130 E2F4 5 HDAC1 SUV39H1 DNMT1 multimolecular complexes mediate the transcription of estrogen receptor alpha  [ **ESR1** ]  in breast cancer.  Ref: 12789259 Oncogene, 2003

**Non-interactive relationship** (e.g. studied together, co-existance, homology, etc.)

- We investigated in vivo occupancy of ER alpha  [ **ESR1** ]  promoter by pRb2 p130 E2F4 5 HDAC1 SUV39 H1  **p300**  and pRb2 p130 E2F4 5 HDAC1 SUV39H1 DNMT1 complexes, and provided a link between pRb2 p130 and chromatin modifying enzymes in the regulation of ER alpha  [ **ESR1** ]  transcription in a physiological setting.  Ref: 12789259 Oncogene, 2003
- In vivo and in vitro binding assays revealed that while 4 hydroxy tamoxifen OHT induced binding of ER alpha  [ **ESR1** ]  to both an AF 1 coactivator complex p68 p72 and  **p300**  and corepressor complexes N CoR SMRT, BBP selectively enhanced the binding to the AF 1 coactivators.  Ref: 12738788 J Biol Chem< MedlineTA>J Biol Chem, 2003
